# Open versus arthroscopic fusion of the subtalar joint: a randomized controlled trial

**DOI:** 10.2340/17453674.2024.42448

**Published:** 2024-12-10

**Authors:** Mark STEGEMAN, Nathalie PRUIJN, Saskia SUSAN, Petra J C HEESTERBEEK, Jan Willem K LOUWERENS

**Affiliations:** 1 Sint Maartenskliniek Research, Sint Maartenskliniek, Nijmegen; 2 Department of Orthopedic Surgery, Sint Maartenskliniek, Nijmegen, the Netherlands

## Abstract

**Background and purpose:**

Our primary aim was to compare the early complication rate (< 6 weeks postoperatively) after open or arthroscopic fusion of the subtalar joint. Secondary outcomes included late complications (> 6 weeks postoperatively), function, pain, and patient satisfaction.

**Methods:**

In this prospective randomized controlled trial, patients listed for subtalar joint fusion were included and randomized for open or arthroscopic fusion. Complications were assessed at scheduled visits at 2 and 6 weeks, 3, 6, and 12 months postoperatively. Functional scores, pain scores, and patient satisfaction were assessed at 3, 6, and 12 months postoperatively, and PROMS at baseline (preoperatively), 3, 6, and 12 months postoperatively. The scores were compared over time between the groups using Fisher’s exact test and linear mixed models.

**Results:**

51 patients were included between 2013 and 2020, of whom 25 were allocated to open and 26 to arthroscopic fusion. 3 early complications (2 sural nerve lesions, 1 infection) occurred in the open fusion group (12%; 95% confidence interval [CI] 3–32) and 3 (2 wound healing problems, 1 screw exchange) in the arthroscopic group (12%; CI 3–31). Late complications included screw removal (n = 5) in the open fusion group versus screw removal (n = 5), non-union (n = 2), bony prominence/calcification removal (n = 1), sural nerve lesion (n = 1), lesion of the calcaneal branch of the tibial nerve (n = 1), complex regional pain syndrome type II (n = 1), and secondary plantar fasciitis (n = 1) in the arthroscopic fusion group. No superiority of arthroscopic over open fusion was found regarding early (P = 1.0) and late complications (P = 0.2), function and pain scores, and patient satisfaction over 12 months

**Conclusion:**

Arthroscopic fusion did not result in fewer early complications compared with open fusion. Secondary outcomes did not differ significantly between the approaches.

Isolated osteoarthritis (OA) of the subtalar joint complex is a painful, disabling condition that limits mobility [1]. OA can be primary or secondary. Secondary OA is seen after trauma, such as calcaneal or talar fractures [2], or, for example, as sequela of talocalcaneal coalitions and subtalar instability [3]. If conservative measures fail, surgical intervention may be considered [1].

For many decades fusion of the subtalar joint through an open procedure has been the standard surgical procedure [4]. Early complications range from 1–30% [3,5] and comprise disturbance in wound healing, infection, and nerve damage [6,7]. Potential late complications are thrombosis, pseudarthrosis, and protruding implants requiring removal [3].

To improve the results and diminish surgical treatment complications of subtalar OA, the technique of arthroscopic subtalar fusion was first published in the late 1990s [4,8,9]. The expectations regarding the arthroscopic (or closed) technique were based on the good results of arthroscopic fusion of the ankle. The number of early complications for the closed technique were reported to be very low [2,3,10-15].

To our knowledge, no randomized controlled trial with sufficient power has been performed comparing complications and function, pain, and satisfaction scores of the open versus arthroscopic fusion of the subtalar joint.

The aim of the present study was primarily to investigate the short-term complications and secondarily to investigate the late complication rate after open and arthroscopic fusion of the subtalar joint. Furthermore, function, pain, and patient satisfaction were studied. The hypothesis was that arthroscopic fusion may encounter fewer short-term complications (primary outcome) than open fusion, because the open technique requires a larger wound with more soft tissue handling.

## Methods

### Study design

In this unblinded randomized controlled trial conducted at the Sint Maartenskliniek (Nijmegen and Woerden, the Netherlands), adult patients with pain and functional impairment caused by primary or secondary subtalar OA were screened for eligibility between 2013 and 2020 from the waiting list for subtalar fusion. Patients were eligible for inclusion when they met the following criteria: isolated subtalar OA, diagnosis primary or secondary OA, at least 6 months’ duration of symptoms, age 18–80 years, less than 15° valgus or less than 5° varus of the hind foot. Exclusion criteria were previous surgery of the subtalar joint, osteonecrosis, diagnosed with rheumatoid arthritis, complex regional pain syndrome, or neurological impairment.

After signing informed consent and before surgery, patients were randomized into the open or arthroscopic fusion group using standard randomization software with allocation ratio 1:1. Randomization lists were created by a colleague researcher at the research department, who was not involved in the study or the treatment of the patients, and were stored safely on a local server, only accessible to qualified personnel. Due to the nature of the surgery, neither patients nor orthopedic surgeons were blinded.

The study is reported according to CONSORT guidelines.

### Surgical technique

Open fusion was performed through a standard lateral incision in order to gain access to the subtalar joint after carefully loosening and retracting the short extensor muscle. After exposure and clearance of the sinus tarsi and the tarsal canal, the remaining cartilage was removed, and the subchondral surface was scaled with an osteotome and awl to create a bleeding cancellous surface to enhance fusion. Subsequently, 2 x 7.3 mm cannulated compression screws were inserted from the calcaneus into the talar body with the hindfoot in correct alignment.

The arthroscopic technique used a posteromedial, posterolateral, and sometimes a third lateral sinus tarsi portal incision around the hind foot. Similar to the open technique, after visualizing the posterior aspect of the subtalar joint, the remaining cartilage was removed and a decorticated cancellous bone surface was created with an acromionizer (Elite Acromionizer Burr 4 mm, Smith & Nephew Dionics, Memphis TN, USA). Thereafter, 2 x 7.3 mm cannulated compression screws were inserted from the calcaneus into the talar body. The postoperative regimen for both groups was 6 weeks of non-weightbearing in a cast, followed by 6 weeks cast with weightbearing allowed. Afterwards a walking boot was offered.

Both surgical techniques were standard of care in our hospital. All arthroscopic fusions were performed by 1 surgeon (MS), who was fellowship trained with over 8 years of experience in arthroscopic surgery. In the open fusion group 20 out of 25 patients were operated on by 1 surgeon (MS) and 5 by other surgeons, all of whom also had extensive experience in foot and ankle surgery.

### Primary outcome

The following early complications (< 6 weeks after surgery) were monitored, specifically: disturbance in wound healing, infection graded according to classification by Sink et al. [16], and nerve damage. Complications were diagnosed by the treating orthopedic surgeon or resident in orthopedic surgery. Since this study had a focus on early (< 6 weeks) complications, complications with an onset in the first 6 weeks with diagnosis after 6 weeks were scored as early complications. Because patients were immobilized in a cast during the first 3 months, wound healing disturbance or subtle nerve dysfunction could only be detected on cast changing outpatient visits. These took place after 2 weeks, around 6 weeks, and 3 months postoperatively or on unscheduled cast changes because of discomfort.

### Secondary outcomes

Secondary outcomes were late complications, and pain and function scores at baseline and 3, 6, and 12 months after surgery. Late complications included complications with an onset between week 6 and 52 postoperatively.

### Questionnaires

Pain and function were measured preoperatively using scores and questionnaires (Numeric Pain Rating Scale [NPRS], American Orthopedic Foot and Ankle Society [AOFAS] hindfoot score, Foot Function Index [FFI], Short Form Health Survey 12-items [SF-12]). After surgery, patients completed these questionnaires (with an additional questionnaire for patient satisfaction) at regular check-up visits at 3, around 6, and 12 months postoperatively.

The NPRS [17] was used to measure pain intensity. Scores ranged from 0–10 points, where higher scores indicate more intense pain.

The AOFAS questionnaire, which focuses on the hindfoot [18], the Hindfoot score, was used as an outcome measure. It has a patient-reported and surgeon-reported section and questions cover pain, function, and alignment. All scores together form a maximum of 100 points. A higher total score represents better outcomes.

The FFI [19] measures the foot pathology impact on pain and activity restriction. Scores ranged from 0 “no pain” to 4 “intense pain” on the pain scale, and from 0 “no difficulty” to 4 “impossible” on the disability scale. To calculate the definitive scale scores, the item scores were summed, divided by the maximum possible sum of the item scores, and then multiplied by 100. The total score was the mean of the scale scores. The scores ranged from 0 to 100; the higher the score, the more pain/disability was present.

The SF-12 [20] measures the patient-reported health state, also known as the quality-of-life score. Scale scores can be calculated for the physical and mental status by summing up the scores from the physical or mental questions and dividing by the maximum physical or mental total score. A higher score indicated a better patient-reported health state.

The patient satisfaction questionnaire included 5 questions concerning the change in daily functioning and pain, and satisfaction with the surgery itself. Answers were scored on a 7-point scale (1–7 points). The scores were summed, with higher scores indicating a better patient satisfaction.

### Other data

Data on body mass index (BMI), age, sex, and smoking were collected.

### Sample size

Sample size calculation was based on the primary outcome: early complications. The proportion of patients with (early) complications for the arthroscopic technique has been reported to be 0% [3,10]. This proportion has been reported to be higher for the open technique, ranging from 1% to 30% [3,5]. As we focus on the total proportion of early complications (and not the type), 30% was therefore taken as the number of expected complications for the open technique and 0% in the arthroscopic group.

Using a sample size calculation for the primary outcome based on comparing 2 proportions [21] in this superiority trial, the number of patients needed per group was 22 (with α = 0.05, β = 0.20). No power calculations were performed for the secondary outcomes. To account for possible dropouts and/or insufficient data quality, the sample size was increased by 20%. This resulted in 26 patients per group, thus 52 in total. These patients were included from 1 center with 2 locations.

### Statistics

Patient characteristics (age, BMI, sex, smoking) and pain and function scores at baseline were summarized using descriptive statistics.

Frequency and percentage were reported for dichotomous variables, with a 95% confidence interval (CI) calculated by Wilson’s approach with continuity correction. Mean and standard deviation (SD) were reported for normally distributed continuous variables, and median and interquartile range (IQR) for skewed continuous variables. Normality of data was inspected using histograms. The number of patients with early and late complications were compared between the groups using Fisher’s exact test.

A scatterplot with trend lines was created to show the operating time per patient in the open and arthroscopic group.

Function, pain, and patient satisfaction scores were compared between the groups over time using linear mixed models, so no patients with incomplete data were discarded. Time and surgery group were set as fixed variables, with intercept, and baseline score as covariate. An interaction between time and surgery group was added to see if scores differed between the groups at 3 months, 6 months, and 12 months postoperatively.

All statistical analyses were performed using SPSS version 21 (IBM Corp, Armonk, NY, USA). A P-value < 0.05 was considered a statistically significant difference. To provide an unbiased comparison, patients who switched from one surgery group to the other were analyzed by the intention-to-treat approach.

### Ethics, registration, data sharing, funding, use of AI, and disclosures

This study was approved by the Ethical Committee “Slotervaartziekenhuis en Reade” (NL44790.048.13) and in accordance with the World Medical Association Declaration of Helsinki. All patients signed a written informed consent form for this study.

This study was registered in the public register of the national ethical committees before the start of the study, but not in a clinical trials registry. No funding was received, no AI was used, and the authors have no conflicts of interest to disclose. Complete disclosure of interest forms according to ICMJE are available on the article page, doi: 10.2340/17453674.2024.42448

## Results

**Figure 1:**
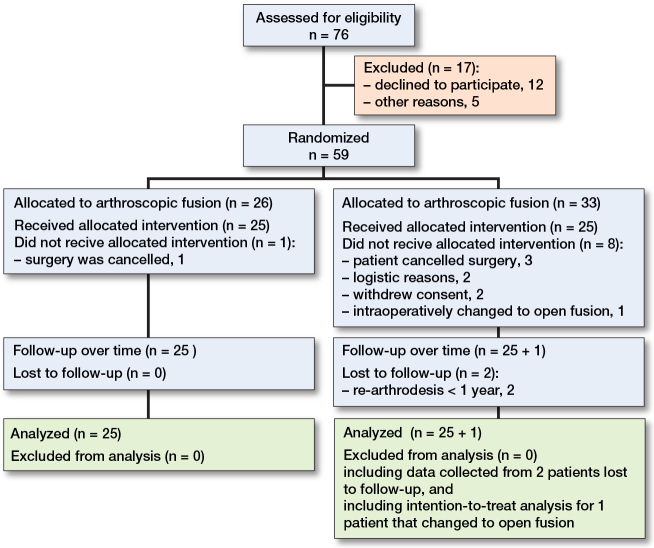
Flowchart of patient inclusion.

76 patients were eligible for inclusion in this study (Figure 1). The exact number of patients screened for eligibility is higher, because at the location in Woerden screened patients were not registered in the first year of this study. 59 patients were randomized and 1 patient in the arthroscopic fusion group was converted intraoperatively to the open technique because of inaccessibility of the joint using the posterior arthroscopic subtalar fusion technique. This patient had posttraumatic arthritis after calcaneal fracture. Because our study protocol included an intention-to-treat analysis, this patient was followed in the arthroscopic group. After arthroscopic fusion, 2 patients were reoperated on due to non-union within 12 months.

51 patients were included, which is 1 less than advised by the sample size calculation. Since inclusion was slow, as can be deducted from the study period from 2013–2021, the authors decided to analyze the available data of 51 patients. This was not expected to affect outcomes significantly.

### Baseline characteristics

Of the 51 included patients, 22 were male and 29 female, with a mean age of 48.5 (SD 15) years. 22 patients were diagnosed with primary OA and 29 had secondary OA of the subtalar joint after calcaneal or talar fracture, tarsal coalitions, clubfoot, and flatfoot (Table 1).

**Table 1:** Baseline characteristics in the open and arthroscopic group

Factor	Open fusion (n = 25)	Arthroscopic fusion (n = 26)
Age, mean (SD)	51 (14)	46 (16)
Body mass index, mean (SD)	28 (5.0)	29 (5.3)
Male/ female sex	13/12	9/17
Smoking		
Yes	15	18
No	7	7
Unknown	3	1

### Outcomes

In the open fusion group, 3 early (12%, CI 3–32) complications were seen versus 3 (12%, CI 3–31) in the arthroscopic fusion group. 5 (20%, CI 8–41) patients experienced late complications in the open group versus 10 patients (38%; CI 21–59) in the arthroscopic fusion group (Table 2). 1 patient in the arthroscopic group suffered a postoperative wound infection in week 7 and was retrospectively considered an early complication. This patient was treated surgically with wound debridement and screw removal in several surgical procedures. Eventually, the arthrodesis consolidated in good alignment and was painless.

**Table 2:** Number of patients with complications in the open and arthroscopic fusion group

Factor	Open fusion (n = 25)	Arthroscopic fusion (n = 26)	P
Primary outcome: early complications			1.0
Wound healing problems (Sink grade 1 [16])	0	2	
Reoperation for screw exchange	0	1	
Sural nerve lesion	2	0	
Infection (Sink grade 3 [16])	1	0	
Secondary outcome: late complications			0.2
Reoperation for screw removal	5	3	
Reoperation for screw removal and sural nerve lesion	0	1	
Reoperation for screw removal and complex regional pain syndrome type II	0	1	
Calcaneal branch of the tibial nerve lesion	0	1	
Non-union^a^	0	2	
Plantar fasciitis	0	1	
Bony prominence/calcification removal	0	1	

^a^ Non-union was diagnosed on CT after 9 months of painful weightbearing, according to the international standard.

**Figure 2:**
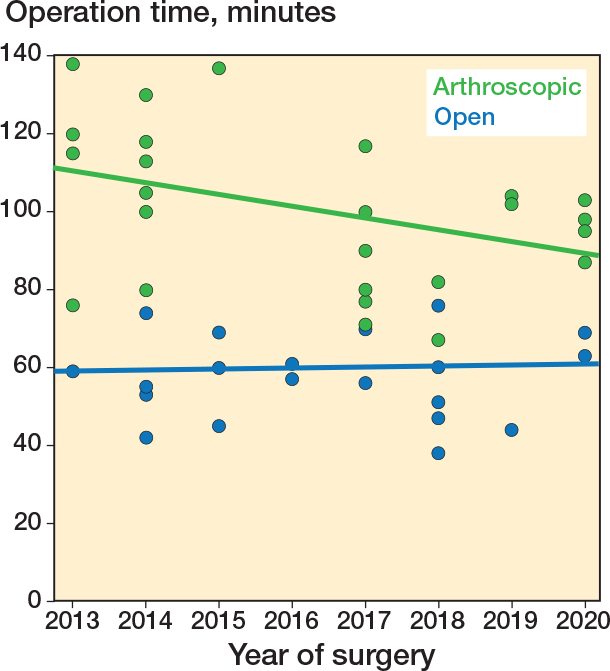
Scatterplot showing the operation time per included patient. Trend lines showing the operation time over the years for the open (blue line) and arthroscopic (green line) group.

The operation time per case is plotted in Figure 2.

The measured NPRS, AOFAS, FFI, SF12, and satisfaction scores at baseline, 3, around 6, and 12 months postoperatively are presented in Table 3. Linear mixed-model analysis showed that there were no statistically significant differences in NPRS, AOFAS, FFI, SF12, and satisfaction scores between the groups over time (Table 4). When an interaction term was added between time and surgery group, SF12 and satisfaction scores were 7.5 (CI 0.8–14) and 5.6 (CI 1.9–9.3) points higher in the open fusion group compared with the arthroscopic group at 6 months postoperatively, respectively. This effect was not present at 3 or 12 months’ follow-up.

**Table 3:** Pain, function, and satisfaction scores for the open and arthroscopic fusion group, measured at different time points. Values are mean (standard deviation) unless otherwise specified

Item	Baseline		3 months		6 months		12 months	
	Open	Arthroscopic	Open	Arthroscopic	Open	Arthroscopic	Open	Arthroscopic
NPRS	6.3 (1.8)	7.1 (1.6)	1.5 (1.7)	2.4 (2.2)	3.5 (2.7)	4.9 (2.7)	3.4 (3.3)	2.7 (2.8)
AOFAS	52 (15)	46 (16)	70 (16)	68 (14)	76 (16)	68 (21)	81 (12)	71 (30)
FFI^a^	43 (32–50)	58 (34–66)	10 (3–23)	13 (10–19)	19 (11–30)	26 (18–42)	17 (7–24)	13 (6–19)
SF12	69 (12)	63 (16)	74 (11)	68 (11)	78 (14)	69 (13)	82 (12)	80 (15)
Satisfaction			26 (6.2)	24 (5.1)	29 (6.9)	23 (7.1)	28 (7.8)	31(5.2)

a Values are presented as median (interquartile range).

Abbreviations: NPRS: Numeric Pain Rating Scale, AOFAS: American Orthopedic Foot and Ankle Society, FFI: Foot Function Index, SF12: Short Form Health Survey 12-items.

**Table 4:** Differences in NPRS, AOFAS, FFI, SF12, and satisfaction scores between the open and arthroscopic group over time, using linear mixed-model analysis with arthroscopic fusion as reference

Item	Estimate (CI)	Std error	P value
NPRS	–0.8 (–2.2 to 0.5)	0.7	0.2
AOFAS	7.2 (–2.1 to 16)	4.6	0.1
FFI	–2.0 (–9.4 to 5.2)	3.6	0.6
SF12	4.2 (–1.7 to 10)	2.9	0.2
Satisfaction	1.9 (–1.3 to 5.2)	1.6	0.2

For abbreviations, see Table 3 and CI = 95% confidence interval

## Discussion

This study is the first prospective randomized controlled trial to compare open with arthroscopic subtalar fusion and aimed to clarify whether a difference exists for the early complication rate, as well as for the late complication rate, function, pain, and patient satisfaction as secondary outcomes. The hypothesis was that arthroscopic fusion would encounter fewer early complications than the open fusion technique. We showed that there was no difference either in early or in late complications, pain, function, and satisfaction scores.

The pain, function, and satisfaction scores found in our study are consistent with the literature, showing good results for both open and arthroscopic subtalar fusion. A challenge remains for surgeons to choose the best surgical procedure for subtalar fusion, as scientific evidence for the preferred technique is absent. Retrospective studies claim good results for both open and arthroscopic subtalar fusion [8,13,14,22,23]. However, a drawback of these studies is that both the groups and accompanying pathologies described are often heterogeneous [3]. Several prospective studies claim excellent results after arthroscopic subtalar fusion and show relatively shorter surgical procedures [2,24,25]. In the present study, operation time for arthroscopic subtalar fusion was found to be significantly longer than for the open technique. Removing all cartilage in the area of the sinus tarsi proved to be time consuming using the posterior arthroscopic subtalar fusion technique [3].

Previous studies of outcomes after open and arthroscopic fusion of the subtalar joint have not been randomized [3,11,25-27]. Only 2 retrospective studies were found comparing open versus arthroscopic subtalar fusion [8,14]. They concluded that there were no specific advantages for either of the 2 techniques regarding complication rates. Rungprai et al. [14] did report a trend towards a higher frequency of implant-related symptoms in the arthroscopic group and a higher frequency of symptomatic sural nerve injury in the open group. Our study showed a similar pattern regarding implant-related problems and sural nerve lesions. They also reported a significantly shorter time to return to work, daily activities, and sport activities for the arthroscopic group compared with the open fusion group [14]. This indicates a smoother early recovery period for the arthroscopic technique, which was expected based on the limited number of early complications found in the literature [2,3,5,10,11]. Our study does not confirm these findings. On the contrary, a higher SF-12 score was found 6 months after surgery for the open fusion group. Even though the SF-12 does not measure function directly, it does provide information concerning a patient’s physical and mental status. Pain and function scores did not differ significantly between the open and arthroscopic fusion groups. In addition, our study reported 8 early and late complications in the open versus 13 in the arthroscopic fusion group over a period of 12 months following surgery, which is not in line with the study of Rungprai et al. [14]. Rungprai et al. [14] described no statistically significant differences in improvement of satisfaction and FFI scores between the open fusion and arthroscopy group at 1- and 2-year follow-up. The present study confirmed earlier results in the literature: no statistically significant differences were found between the open and arthroscopic fusion group regarding pain, activity, and satisfaction scores at 12 months’ follow-up.

The operative time was found to be significantly longer in the arthroscopic group than in the open technique group although a slight decreasing trend was observed throughout the study, most likely due to the learning curve, which is in accordance with earlier studies [2,14].

### Clinical relevance

Our study provides insight into the advantages and disadvantages of arthroscopic versus open techniques but has not led to change in the treatment of subtalar OA in our practice. In our practice, the open technique is mostly performed, and arthroscopic fusion only when indicated based on scarring/soft tissue problems over the sinus tarsi, combined with posterior intervention, e.g., removal of a symptomatic os trigonum or posttraumatic ossicles. In our experience, the open technique provides a fast and safe approach to the subtalar joint. In addition to the longer operation time, a disadvantage of the posterior arthroscopic subtalar fusion technique is the prone position of the patient, which is time consuming and potentially problematic from an anesthesiologic point of view.

### Strengths

This RCT was performed with 1 surgeon performing all arthroscopic and most open technique surgeries in 1 center, minimizing bias, providing a direct prospective comparison, and the focus was placed on a homogeneous group of patients suffering subtalar OA.

### Limitations

We had a prolonged inclusion period, which was caused by strict in- and exclusion criteria. Because of the strictly selected population, questions may arise regarding generalizability of the results. 30% difference in expected complications between the open and arthroscopic fusion group was probably overrated, which was estimated and used for the sample size calculation. A risk of increased type II error is therefore present. This study was registered before the start of the study in the public register of the national ethics committees rather than in a better known trial register. At the time of the start of the study, the policy of the department was different and pre-registration in another trial register was not an obligation, as it is nowadays. We are confident that the results of this study are not affected by this, as all staff involved in this study strictly adhered to the protocol and planned analyses, as uploaded in the public register before the start of the study.

### Conclusion

We did not find any difference in early complications, late complications, and pain, function, or satisfaction scores. Advantage of arthroscopic subtalar fusion over open fusion could not be established in this study.

## Notes

MS wrote the study protocol and supervised the planning of the study. SS was the research nurse and took care of patient eligibility, informed consent, and source data. PH and NP performed data analyses. MS and NP wrote the article. MS, PH, JWL, NP, and SS reviewed the draft article. NP edited the draft into the final article and took care of journal submission.

Handling co-editors: Paul Gerdhem and Philippe Wagner

*Acta* thanks Anna Eva Sprinchorn and other anonymous reviewers for help with peer review of this manuscript.
